# Wrist actigraphic approach in primary, secondary and tertiary care based on the principles of predictive, preventive and personalised (3P) medicine

**DOI:** 10.1007/s13167-021-00250-5

**Published:** 2021-08-06

**Authors:** Jens Acker, Olga Golubnitschaja, Antje Büttner-Teleaga, Kneginja Richter

**Affiliations:** 1 Clinic for Sleep Medicine, Zurzach Care, Badstrasse 33, 5330 Bad Zurzach, Switzerland; 2 Predictive, Preventive and Personalised (3P) Medicine, Department of Radiation Oncology, University Hospital Bonn, Rheinische Friedrich-Wilhelms-Universität Bonn, Venusberg-Campus 1, 53127 Bonn, Germany; 3 Institute of Cognitive Science, Woosuk University, Samnye-up, South Korea; 4 Department of Psychiatry, University Witten-Herdecke, Witten, Germany; 5 Sleep Ambulance, University Clinic for Psychiatry and Psychotherapy, Paracelsus Medical University, Nuremberg, Germany; 6 Institute for E-Counselling, Technical University for Applied Sciences Nuremberg Georg Simon Ohm, Nuremberg, Germany; 7 Faculty for Medical Sciences, University Goce Delcev, Stip, Macedonia

**Keywords:** Actigraphy, Predictive preventive personalised medicine (PPPM/3PM), Sleep quality tool, Sleep disturbance, Sleep disorder, Chronobiology, Circadian rhythm, Insomnia, Sleep–wake rhythm, Exposition to light, Mental health, Primary secondary tertiary care, Patient stratification, Targeted prevention, Tailored treatment, Individualised patient profiling, Mood, Pain, Fatigue, Post-traumatic stress disorder, Motor activity, Behavioural patterns, Military, Sport medicine, Performance enhancement, Personalised medication, Cancer, Depression, Dementia, ICU, COVID-19 pandemic, Individual outcomes, Traffic safety, Health policy, Recommendations

## Abstract

**Abstract:**

Sleep quality and duration as well as activity-rest-cycles at individual level are crucial for maintaining physical and mental health. Although several methods do exist to monitor these parameters, optimal approaches are still under consideration and technological development. Wrist actigraphy is a non-invasive electro-physical method validated in the field of chronobiology to record movements and to allow for monitoring human activity-rest-cycles. Based on the continuous recording of motor activity and light exposure, actigraphy provides valuable information about the quality and quantity of the sleep–wake rhythm and about the amount of motor activity at day and night that is highly relevant for predicting a potential disease and its targeted prevention as well as personalisation of medical services provided to individuals in suboptimal health conditions and patients. Being generally used in the field of sleep medicine, actigraphy demonstrates a great potential to be successfully implemented in primary, secondary and tertiary care, psychiatry, oncology, and intensive care, military and sports medicines as well as epidemiological monitoring of behavioural habits as well as well-being medical support, amongst others.

**Prediction of disease development and individual outcomes:**

Activity-rest-cycles have been demonstrated to be an important predictor for many diseases including but not restricted to the development of metabolic, psychiatric and malignant pathologies. Moreover, activity-rest-cycles directly impact individual outcomes in corresponding patient cohorts.

**Targeted prevention:**

Data acquired by actigraphy are instrumental for the evidence-based targeted prevention by analysing individualised patient profiles including light exposure, sleep duration and quality, activity-rest-cycles, intensity and structure of motion pattern.

**Personalised therapy:**

Wrist actigraphic approach is increasingly used in clinical care. Personalised measurements of sedation/agitation rhythms are useful for ICU patients, for evaluation of motor fatigue in oncologic patients, for an individual enhancement of performance in military and sport medicine. In the framework of personalised therapy intervention, patients can be encouraged to optimise their behavioural habits improving recovery and activity patterns. This opens excellent perspectives for the sleep-inducing medication and stimulants replacement as well as for increasing the role of participatory medicine by visualising and encouraging optimal behavioural patterns of the individual.

## Introduction

Sleep quality and duration are crucial for maintaining physical and mental health [1, 2] as well as for individual outcomes in a spectrum of suboptimal health conditions, external and internal risk factors with health adverse effects [3–6] as well as pathologies under treatment [7–10].

Contextually, individualised sleep quality monitoring is essential to analyse individual sleep patterns, to predict sleep disorders and associated pathologies followed by targeted prevention and treatments tailored to the personalised patient profile [9, 11–14]. This approach is conform with principles of predictive, preventive and personalised medicine (PPPM/3PM) [15].

In the field of sleep medicine, wrist activity is used for estimating sleep parameters over periods of days to weeks [16]. Devices developed in the branch allow for monitoring over a long period of time in the patient’s natural environment. The actigraphy approach has been used for nearly 60 years to study sleep and determine 24-h motor activity [17]. Corresponding devices can be worn on the wrist, ankle or waist. Usually, actigraphy is completed with sleep logs and most used for measuring sleep latency (SL), total sleep time (TST), wake after sleep onset (WASO) and sleep efficiency (SE; corresponds TST/time in bed TIB).

Investigating sleep quality at home with objective means like actigraphy is supported by growing evidence on the importance of sleep–wake rhythm organisation and sleep duration per se [18, 19].

In recent years, actigraphy has been increasingly used in clinical settings. Modern medical actigraphs have achieved increased accuracy and reliability with improved piezoelectric motion sensors, lithium batteries and extended memory capabilities [20].

With the increasing availability of inexpensive actigraphy in wearables, promising applications are opening in the field of sleep medicine and in predictive, preventive and personalised medicine. These will be presented and discussed in the following.

## Actigraphy in the field of sleep medicine

### Increasing recognition of actigraphy in the field of sleep medicine

In 1995, the ASDA provided first recommendations for the use of actigraphy in the clinical assessment of sleep disorders [21]. Actigraphy was labelled as a complementary method for documenting multiday rest-activity patterns [22] and later on used both in the field of sleep medicine and sleep research.

The Standards of Practice Committee of the American Academy of Sleep Medicine (AASM) updated the “Practice Parameters for Actigraphy” in 2007 [23] and 2018 [24], leading to increased prominence in the field of clinical sleep medicine.

To date, the use of actigraphy is recommended in the following sleep disorders or circadian rhythm sleep–wake disorders [25]:

- to estimate sleep parameters in adult patients with insomnia disorder (conditional)
- in the assessment of paediatric patients with insomnia disorder (conditional)
- in the assessment of adult patients with circadian rhythm sleep–wake disorder (conditional)
- in the assessment of paediatric patients with circadian rhythm sleep–wake disorder (conditional)
- integrated with home sleep apnea test devices to estimate total sleep time during recording (in the absence of alternative objective measurements of total sleep time) in adult patients suspected of sleep-disordered breathing (conditional)
- to monitor total sleep time prior to testing with the multiple sleep latency test in adult and paediatric patients with suspected central disorders of hypersomnolence (conditional)
- to estimate total sleep time in adult patients with suspected insufficient sleep syndrome (conditional)

The use of actigraphy in place of electromyography for the diagnosis of periodic limb movement disorder in adult and paediatric patients is not recommended anymore.

Obstructive sleep apnea (OSA) detection with actigraphy and long-term monitoring of behavioural change is a prominent example in sleep medicine. OSA is a common disorder worldwide that is still often not recognised or treated [26]. The use of actigraphy in combination with conventional sleep diagnostics has been suggested for more than 15 years [27]. In the case of limited access to sleep medicine care, a combination of actigraphy and polygraphy can be used to clarify respiratory disorders and the associated sleep fragmentation [28, 29]. Actigraphy can detect the main sleep period in patients with OSA well, but the differentiation from waking phases remains problematic [30]. In a recent proof of principle study, a sufficient match with polysomnography was found with further developed evaluation algorithms using artificial intelligence based on actigraphy and respiratory excursion data. Thus, with a significantly simplified recording technique, a greatly simplified diagnosis of sleep-related respiratory disorders could be established [31]. Drowsiness is a common symptom of OSA that can resolve with successful treatment. Interestingly, OSA patients without obesity showed a significant increase in activity as measured by wrist actigraphy, whereas overweight patients did not [32].

### Recommendations by the international classification of sleep disorders (ICSD-3)

The new version of the international classification of sleep disorders (ICSD-3) [33] recommends the use of actigraphy in numerous diagnostic groups:

- central disorders of hypersomnolence (narcolepsy, hypersomnia)
- behaviourally induced insufficient sleep syndrome (BISS)
- long sleeper (normal variant)
- circadian rhythm sleep–wake disorders
  - delayed sleep–wake phase disorder
  - irregular sleep–wake rhythm disorder
  - non-24-h sleep–wake rhythm disorder
  - shift work disorder
  - circadian sleep–wake disorder not otherwise specified

### An issue of reimbursement for the clinical implementation of actigraphy

There is a discrepancy between the recommendation to implement actigraphy on one hand but still problematic reimbursement on the other hand [34, 35]. It is evidently a bottle-neck in the clinical application of actigraphy that in most countries, the reimbursement of actigraphy is not implemented [36] or regulated inconsistently [37].

## Actigraphy application in the broader focus of sleep medicine

Everyday activity patterns play an important role in the diagnostic and treatment of sleep disorders. When measuring total sleep time under free-living conditions, it is important to evaluate objective measures that are less susceptible to reporting biases than sleep logs.

### Actigraphy is instrumental to quantify sleep duration

Actigraphy could provide greater degrees of objectivity of sleep measurements than subjective or parental reports. In contrast with the growing scientific and clinical interest in this method, age-appropriate and gender-based data are still missing [38, 39]. Additionally, customising the standard actigraphy settings may be necessary in certain populations [40]. Recently studies found that objective actigraphy-based sleep data were not well correlated with subjective sleep quality [41].

Other studies with depressed insomniacs showed a superior actigraphy agreement with polysomnography data compared to sleep diaries [42].

### Long-term monitoring of sleep–wake data with traditional actigraphy device

The potential health risk of sleep deprivation and long-term sleep curtailment has been repeatedly discussed in recent literature [43–46].

Motor activity recording by wrist actigraphy in a famous sleep researcher over period of 33 years shows the great impact society exerts on the structure of the sleep–wake cycle [47]. The weekly 1-h sleep prolongation on weekends vanished post-retirement.

The phenomenon of social jetlag is linked to several adverse health outcomes including obesity and cardiometabolic risk [48]. Actigraphy provides objective behavioural measures of sleep patterns that are not influenced by retrospective reporting bias, in contrast to sleep logs for example.

### Insufficient sleep duration and circadian rhythm disruption

Insufficient sleep per se is the first cause for several adverse health consequences [49]. Impairments in physiological functioning, cognitive deficits and emotional effects ranging from mood instability to depressive symptoms may be caused by insufficient rest times. Diagnostic criteria for behaviourally induced insufficient sleep syndrome (BISS) according to ICSD-3 include an urge to sleep, daytime lapses into sleep for 3 months and a low sleep duration according to age [33]. Some authors proposed to define sleep insufficiency as a potential non-communicable disease [50].

Several studies establish a link between fragmented sleep, sleep deprivation measured by actigraphy and increased obesity susceptibility. Today, therefore, addressing sleep deprivation in the general population is a major challenge for the future for all industrialised nations [51].

Epidemiological studies indicate a strong association between the circadian clock and human health [52]. The disruption of complex interacting metabolic processes, for example through shift work, results in an increased risk of metabolic syndrome, obesity and consequently diabetes [53], amongst others. Corresponding algorithms allow for analysis of sleep–wake patterns [54].

### Discriminating between sleepiness and fatigue

In the borderland of fatigue, sleepiness and tiredness, discriminating behaviourally induced sleepiness from disease-related fatigue can be difficult. Behaviourally induced insufficient sleep syndrome (BISS) can be unmasked by combining sleep diaries and actigraphy. Sleepiness can be objectified by using a standardised procedure for measuring sleepiness called multiple sleep latency test (MSLT) [55].

### Actigraphy as a tool for keeping a control over safety in a spectrum of areas

Per evidence, sleep disorders are underestimated and underdiagnosed in the primary care [56]. Therefore, actigraphy is suitable to uncover reporting bias [35]. In some studies, actigraphy was used for monitoring adherence to study procedures [57]. Others used actigraphy to monitor and verify compliance with the sleep restriction treatment recommendation [58]. Assessment of functional recovery in paediatric patients perioperative care could be an important addition for improving individual care [59]. The correct diagnosis and treatment of daytime sleepiness remains a relevant contribution of sleep medicine to the prevention of the issue-related accidents, e.g. from the perspective of the traffic safety as well as professional and private safety [60].

## Actigraphy application opportunities beyond the sleep medicine

Recently, several groups reported use of actigraphy in different areas of application.

### Monitoring of sedation/agitation levels in intensive care unit (ICU)

Accelerometer-based devices, placed on the nondominant wrist, were correlated to measures of a proven sedation/agitation scale. The authors found a strong correlation between the sedation/agitation levels and the actigraphy data and concluded that actigraphy may have the potential to be used as a tool for automated objective real-time monitoring of sedation/agitation levels for patients in ICUs [61]. They proposed that actigraphy data could be presented at bedside, freeing up nursing time for other patient care activities and allow monitoring the patients in absence of the nursing staff. Future research is needed in this area, before decision-making in critically ill populations can be supported [62].

### Monitoring activity to estimate energy expenditure

The WHO started to include regular physical activity (PA) amongst the global recommendations related to health and non-communicable disease prevention [63]. In most developed countries, PA levels stay far behind the given recommendations in terms of intensity and duration. Activity behaviour amongst middle-aged German adults for example stays far behind the WHO recommendations in > 85% of the subjects [64].

Actigraphs could be used for estimation of energy expenditure, as they save information about frequency and intensity of PA. Problematic is the missing integration of type of PA [65]. Modern prevention recommendations aim at changing health behaviour and in particular increasing physical activity [66]. In fact, the objective change in behaviour is rarely tracked and objectified. Actigraphy offers the possibility to verify the implementation of expert recommendations. Participants in prevention programmes can receive visualised feedback on the extent to which recommendations are implemented in everyday behaviour.

### Cancer-related fatigue and poor sleep in organ transplant recipients

Sleep–wake rhythm disturbance in hospital environment is highly relevant for the degree of cancer-related fatigue. For example, objective actigraphic data showed a greater inter-daily instability of sleep–wake rhythm in paediatric patients with acute lymphoblastic leukaemia. Corresponding intervention options aimed to improve sleep-hygiene and individual physical activity are highly desirable to be developed based in the actigraphic approach [67].

Poor sleep is common amongst organ recipients. To improve post-transplant outcomes, actigraphy can be used to identify individuals with poor sleep quality and initiate targeted personalised therapeutic interventions.

### Dementia and MCI

The severity of sleep–wake rhythm disorders is related to the severity of neuropsychological dysfunction in patients with mild cognitive impairment (MCI) [68], and it is widely accepted that sleep problems are common amongst patients suffering from the MCI [69]. To this end, the prevalence of sleep disturbance may be intermediate between normal ageing and dementia. Thus, alterations in the circadian structure may be early indicators for age-related cognitive decline. In a pilot study, the delay of the acro-phase (time a rhythm peaks) was found in a subgroup of patients that showed cognitive decline, while the general parameters of sleep did not differ significantly. It has been concluded that changes in circadian rhythm may be a precursor of clinically relevant cognitive decline in older adults [70].

### Military medicine

In military personnel with post-traumatic stress disorder, an impaired nocturnal melatonin secretion has been demonstrated, compared to controls [71]. For this patient cohort, individualised therapy algorithms can be established and controlled with the help of actigraphy.

### Monitoring individual sleep quality under pandemic-related movement restrictions

During the COVID-19 pandemic, analysis of cloud-based activity data has shown significant changes at the population level in terms of sleep duration and bedtime [72].

Due to the existing baseline restrictions in many countries, physical activities have decreased significantly with average sleep times increasing [73]. The social restrictions led to robust changes in mid-sleep time [74], indicating a massive sleep deficit under pre-pandemic conditions.

To this end, actigraphic measurements might be useful to improve day-night rhythms individually and to provide recommendations under lasting pandemic conditions.

## Actigraphy provides significant advantages for the 3PM approach

A widespread use of actigraphy could provide sleep disturbances/deficits risk assessment for primary care. Actigraphy enables an objective recording of everyday behaviour patterns that advances a descriptive survey approach [35, 75]. It can fill gaps in the report of nocturnal behaviour and provide substantial information additionally to sleep diaries and interviews [76].

Detailed tracking of patient routines in the home environment can significantly improve an individualised patient profiling approach contributing to the paradigm change from reactive to predictive, preventive and personalised medicine [77].

To this end, the great advantage of the actigraphy is that it can provide valid information about nightly waking hours and sleep patterns, particularly in case of children, dementia patients, patients with communication handicaps and malcompliance [76].

### Actigraphy is instrumental for participatory medicine—increasing level of motivated prevention and acceptance by the patient

In several prospective studies, actigraphy was used over the 24-h period to record daily activity versus inactivity phases. Increased mortality for subjects with a prevalence of sedentary patterns was the most important finding of this research [78]. To this end, actigraphy might be the best tool to communicate the data to the patient illustrating corresponding recommendations to motivate them for improved behavioural patterns [79, 80].

### Innovative concepts for extracting information from conventional actigraphy data

New concepts of the actigraphy data processing allow for extracting information over the 24-h period of time [81]. The focused observation of inactivity during sleep phases and the calculation of intra-individual activity dynamics over the course of the night enable sleep structure and sleep dynamics characterisation with relatively simple means.

Furthermore, in countries with a restricted availability of polysomnography diagnostics, the innovative and highly cost-effective actigraphy approach can be utilised to predict sleep disorders followed by the targeted intervention such as morning light exposure combined with physical activity leading to the significantly improved symptoms of delayed sleep phase syndromes without further medication [82].

### Barriers in producing certified devices

The production of wearables is currently an unregulated space; a vast majority of the devices are not approved as regular medical devices and are thus categorised as wellness products [83]. Currently corresponding initiatives go for certification of digital health software, and manufacturers look for partnering in science and with relevant clinics to trigger production of certified medical devices [84].

### Actigraphy in psychiatry supports principles of 3P medicine

Well-being in the population can be significantly improved by reducing sleep disorders [85]. Further, for patients suffering from sleep disorders but without depression history, the risk of developing a depression can be significantly reduced by early detection and timely treatment of insomnia [86]. According to recently published statistics, insomnia is an independent predictor for the development of anxiety disorders, alcohol abuse and psychotic disorders, amongst others [87, 88]. Contextually, individualised prediction and targeted prevention of sleep disorders would sufficiently contribute to the field of psychiatry that demands further research.

Early detection of sleep–wake rhythm disturbances and insomnia is supportive for a cost-effective prevention and personalised treatment of the related psychiatric disorders.

Actigraphy can provide insights into actual nocturnal sleep fragmentation during ongoing therapy, as it has been demonstrated for the post-traumatic stress disorder. In a sample of patients with schizophrenia-spectrum disorders, reduced motor activity (measured by wrist actigraphy) was a marker for functional deterioration in the course of the disease. In patients with multiple episodes, motor activity was higher during the first episode [89]. The promotion of physical exercise in this patient group may be supported using objective data, generating a dynamic feedback for patients [90].

Moreover, healthy population may significantly benefit from those preventive measures aiming at the improved sleep quality protecting individuals against related psychiatric and other pathologies [91].

## Individualised actigraphy application—case studies

**Fig. 1:**
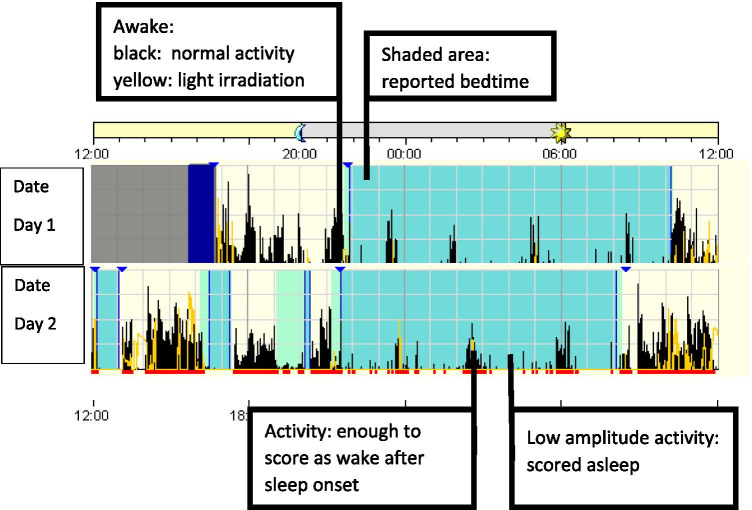
Exemplified general recommendations for interpretation of wrist actigraphy; general process: example of an ambulatory device with piezoelectric movement measurement. Device is worn for multiple 24-h periods in the natural environment (e.g. 7–14 consecutive days/nights). Usually, a sleep diary is kept parallel to the actigraphy. The information from the sleep diary and the visual markers of the actigraphy (here: blue triangles) are used for data adjustment and support the discussion of the findings with the patient. Technical procedure: device specific software is used to score and analyse the data. Activity counts are translated into epochs (e.g. 30 s/1 min) and algorithm is used to determine if each epoch is “sleep” or “wake”. Depending on algorithm the scoring is based on index epoch plus epochs before and after the index epoch. Data interpretation: shaded area shows reported bedtime (main sleep opportunity). Visible is a longer main sleep phase and several short daytime sleep phases. By using the scoring algorithm, the possible sleep time can be delimited from nocturnal movement phases (activity in black, partly with light irradiation in yellow). Daytime sleep episodes and general activity patterns can be estimated. General daily activity can be estimated and objectified; in particular longitudinal changes of activity patterns can be intra-individually determined

General recommendations for the interpretation of wrist actigraphy are exemplified and detailed in Fig. 1.

### Primary care: suboptimal health conditions prior to clinical manifestation of a disease

Long periods of bed rest and low levels of physical activity are associated with increased cardiovascular risk [92] and mortality [93]. It is extremely difficult to comprehensively assess the daily habits of patients in a short period of time in a GP’s brief contact. In particular, the survey of regular physical activity according to the recommendations of the World Health Organisation remains a challenge [63]. The activity behaviour amongst middle-aged adults remains highly insufficient [64].

The 45-year-old patient was referred by his general practitioner with the core symptoms of therapy-resistant sleep disturbance and non-restorative night sleep. The focus of the problem at the time of referral was the poor quality of sleep; attempts at improvement with sedating substances were unsuccessful.

The 14-day actigraphy initiated after taking the patient’s history showed a predominantly sedentary lifestyle with almost complete avoidance of physical activity. Significant deconditioning was present, and physiotherapy was indicated.

The patient underwent a cycle of outpatient medical training therapy and reported an improvement in his daily well-being as well as a decrease in the sleep problem that initially led to his presentation.

The use of actigraphy in this case enabled the support of sustainable behaviour change. The patient’s original focus on a health disorder (“sleeping sickness”) could be expanded to a broader understanding of the concept of health (“I can be more active in my everyday life and live healthier”).

Inadequate and low daytime activities lead to a reduction in sleep pressure, which is needed for stable and subjectively satisfactory night-time sleep [94]. As a result, the subjective patient complaints of “insomnia” often lead to an increasing prescription of sleeping pills with age [95]. There is an evident need to increase physical activity in several sub-populations, e.g. suffering from sedentary lifestyle. To this end, development of actigraphy with gamification approaches can be a way to support sustainable improvements in behavioural patterns that is challenging [96].

**Fig. 2:**
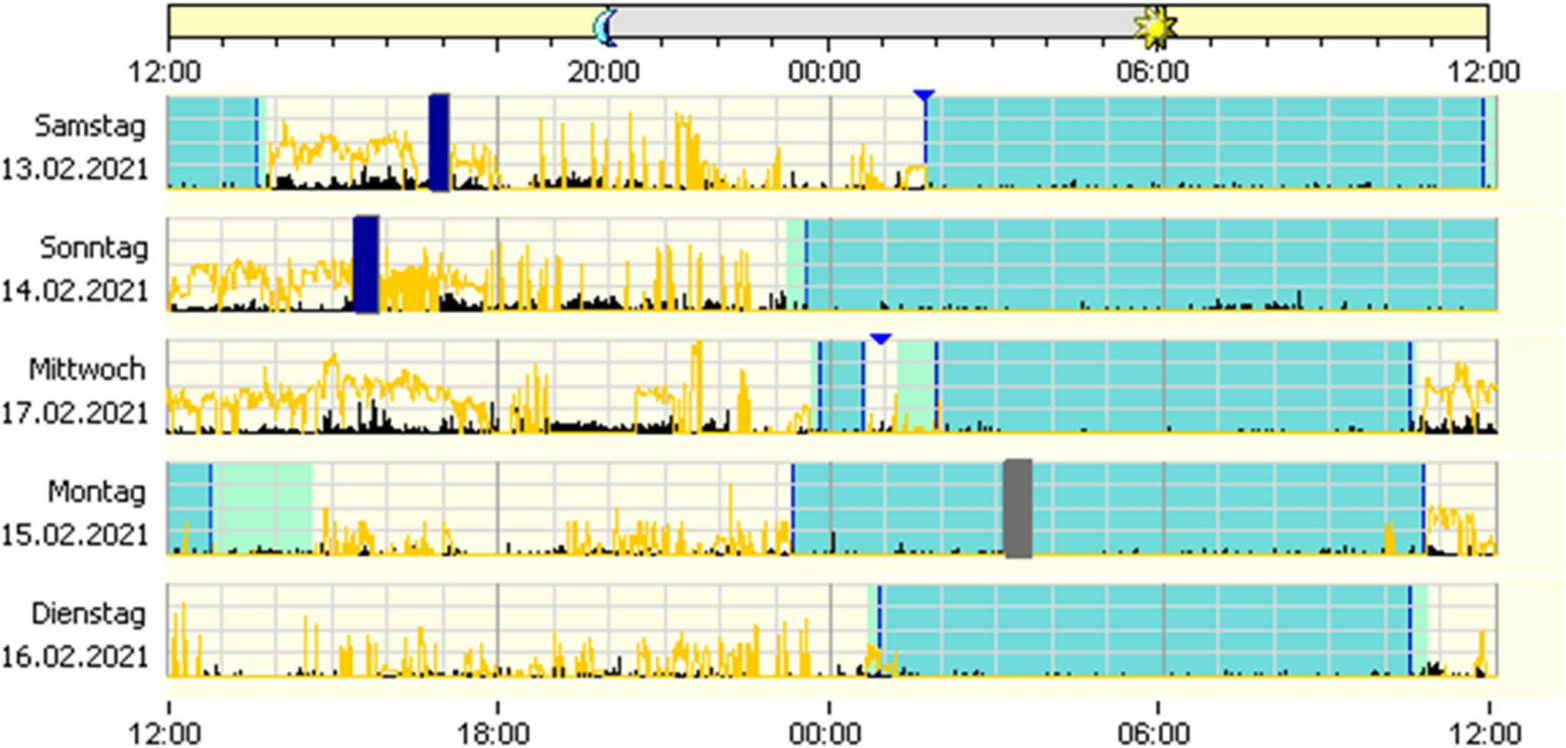
Application of actigraphy to clinophilia/sedentary behaviour: section of a 2-week actigraphy with significantly prolonged bed-lying phase, decreased daytime activity, several days without significant daytime light exposure. The dark areas indicate the patient’s bed phase, black indicates motor activity, yellow light exposure

Figure 2 exemplifies clinophilia/sedentary behaviour (long bedtime, low physical activity, low light exposure).

### Secondary care (to avoid complications and cascading pathologies)

A careful family history opens additional perspectives in the context of therapy, as does the use of objective diagnostics, which can be of importance for therapy [97].

A 65-year-old pensioner was referred by his family doctor because of his sleep disturbances attributed to a decrease of the benzodiazepine effect of the hypnotic therapy prescribed for many years.

After a prolonged actigraphy was performed over 4 weeks, an unusual pattern of sleep fragmentation was revealed. An extended family history was taken to adjust the intended behavioural therapy for insomnia (CBT-I).

Considerable computer gaming activity and imminent indebtedness became evident. Specialist psychiatric treatment for the underlying disorder “gambling addiction” was initiated. A rapid change in the patient’s behaviour has been achieved through 24-h support by family members. Medium-term psychiatric therapy was not desired by the patient.

The relationship between insomnia symptoms and obsessive–compulsive symptoms was also postulated in the context of the corona pandemic [98]. In the presented patient case, however, no increased psychological stress was subjectively reported due to the corona crisis.

Considering particularities characteristic for adolescents, clear associations have been demonstrated between extensive media consumption, playing video games and the development of sleep disorders [99, 100]. Prolonged media consumption can cause or complicate the course of psychiatric illnesses through sleep time reduction and social isolation [101]. Consequently, there is a need to establish objective measurement standards for sedentary behaviour, light exposure and light, moderate and vigorous activity [102]. More advanced forms of actigraphy can then make an important contribution to the improved behavioural patterns in these subgroups. This is particularly important because of the association of sedentary behaviour with metabolic syndrome, arterial hypertension and colorectal cancer [103–105].

**Fig. 3:**
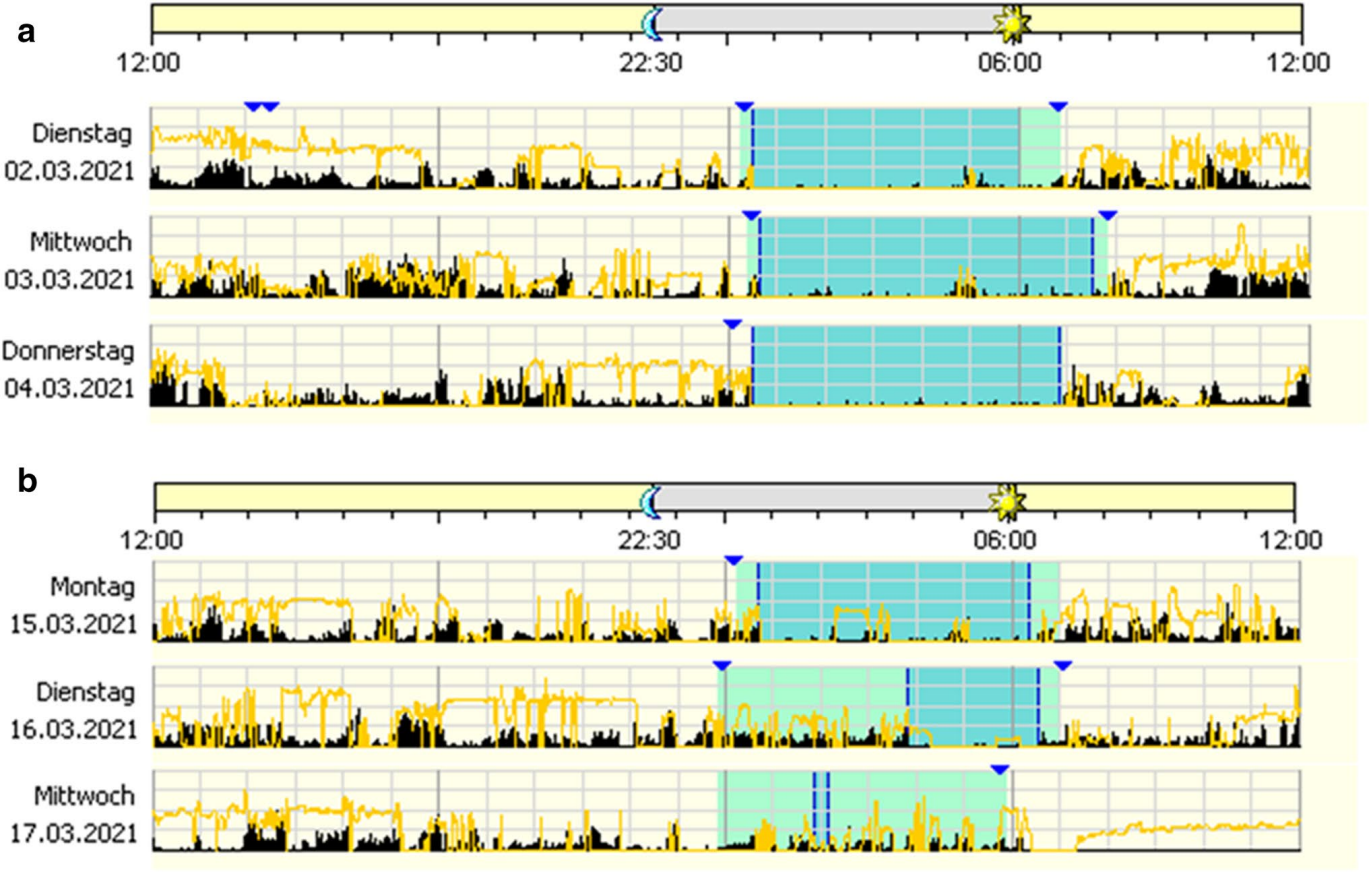
**A** Normal sleep phase: recorded phase with normal sleeping behaviour, in the presence of the partner. The dark areas indicate the patient’s bed phase, black indicates motor activity, yellow light exposure. **B** Nocturnal computer use and massive fragmentation of the main sleep phase: decompensated gambling addiction in the further course in the absence of the family members. The dark areas indicate the patient’s bed phase, black indicates motor activity, yellow light exposure

Figure 3 demonstrates normal sleep phase (A) and excessive media use (B) in a person with unknown gambling addiction.

### Tertiary care

Fifty-seven-year-old patient with chronic pain syndrome on escalated opiate therapy was referred because of significant daytime fatigue combined with pain and sleep disturbances.

Actigraphy shows a predominantly abolished circadian rhythm with rest phases distributed throughout the day. Painkillers and sedative substances are taken in medium to high doses only when needed. General practitioner prescription schemes are no longer observed. The patient was hospitalised for reduction of pain medication and readjustment of medication.

In our case, the use of the actigraphy method in cooperation with the pain physician led to a changed view of the subjective patient information regarding pain experience and prevented the further escalation of pain therapy.

From a sleep- and chronomedical point of view, light therapy in the morning (breakfast on the balcony) was recommended, as well as retarded melatonin added to the basic therapy in the evening [106].

Finally, the establishment of an outpatient service led to a pain therapy based on medical and pharmacological criteria [107]. With a reduced total medication, an improved patient condition has been achieved.

The use of actigraphy in combination with pain diaries and standardised assessments is recommended for chronic pain patients [108]. The use of polysomnography is reserved for the search for primary sleep disorders and is associated with a much higher consumption of resources.

**Fig. 4:**
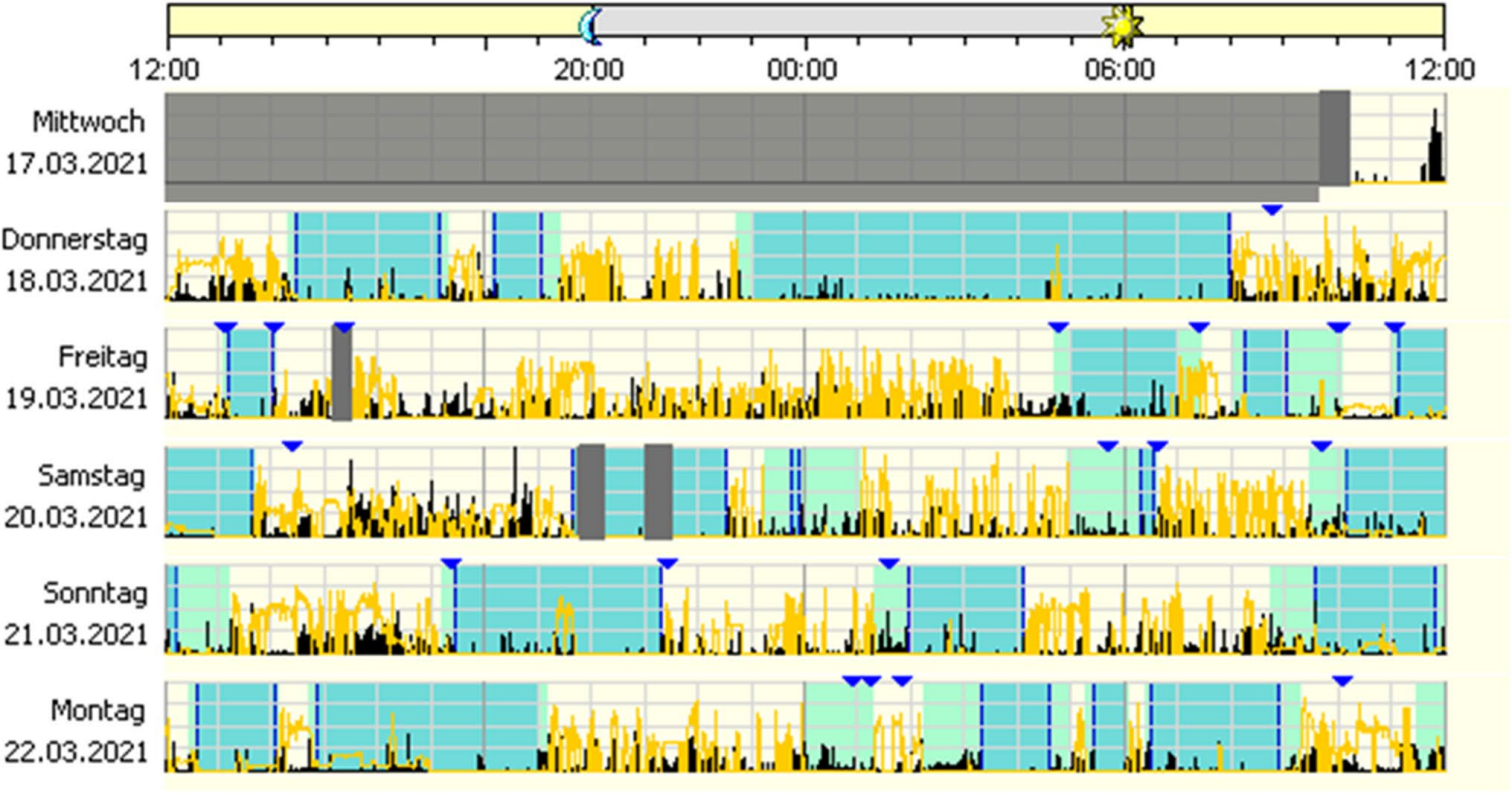
Irregular sleep–wake rhythm disorder (ISWRD): the section of a 3-week actigraphy shows an unidentifiable main resting phase, various sleep attempts occur (indicated by the triangles marking the beginning and end of a sleep attempt, markings are often forgotten). The disorganised supply of pain medication and sedative medication was problematic. From the actigraphy, it becomes clear that GP therapy recommendations on intake times probably cannot be implemented

Figure 4 highlights irregular circadian sleep–wake cycle in a chronic pain syndrome.

### Help in adolescence: a psychiatric patient with suspended day-night structure

Adolescents frequently complain of academic and personal difficulties in psychiatric outpatient clinics, which often leads to a standard psychiatric diagnosis. Especially in the presence of relevant sleepiness and fatigue, the use of actigraphy can provide complementary and high relevant information towards circadian rhythm disorders [109].

Taking into account the principles of chronomedical therapy and the two-process model of sleep [94], an improvement of the day-night rhythm can be achieved through regular eating times, improvement of the activity level with simultaneous light exposure and melatonin administration [110].

After normalisation of the day-night rhythm, the patient has been admitted to long-term psychiatric therapy.

**Fig. 5:**
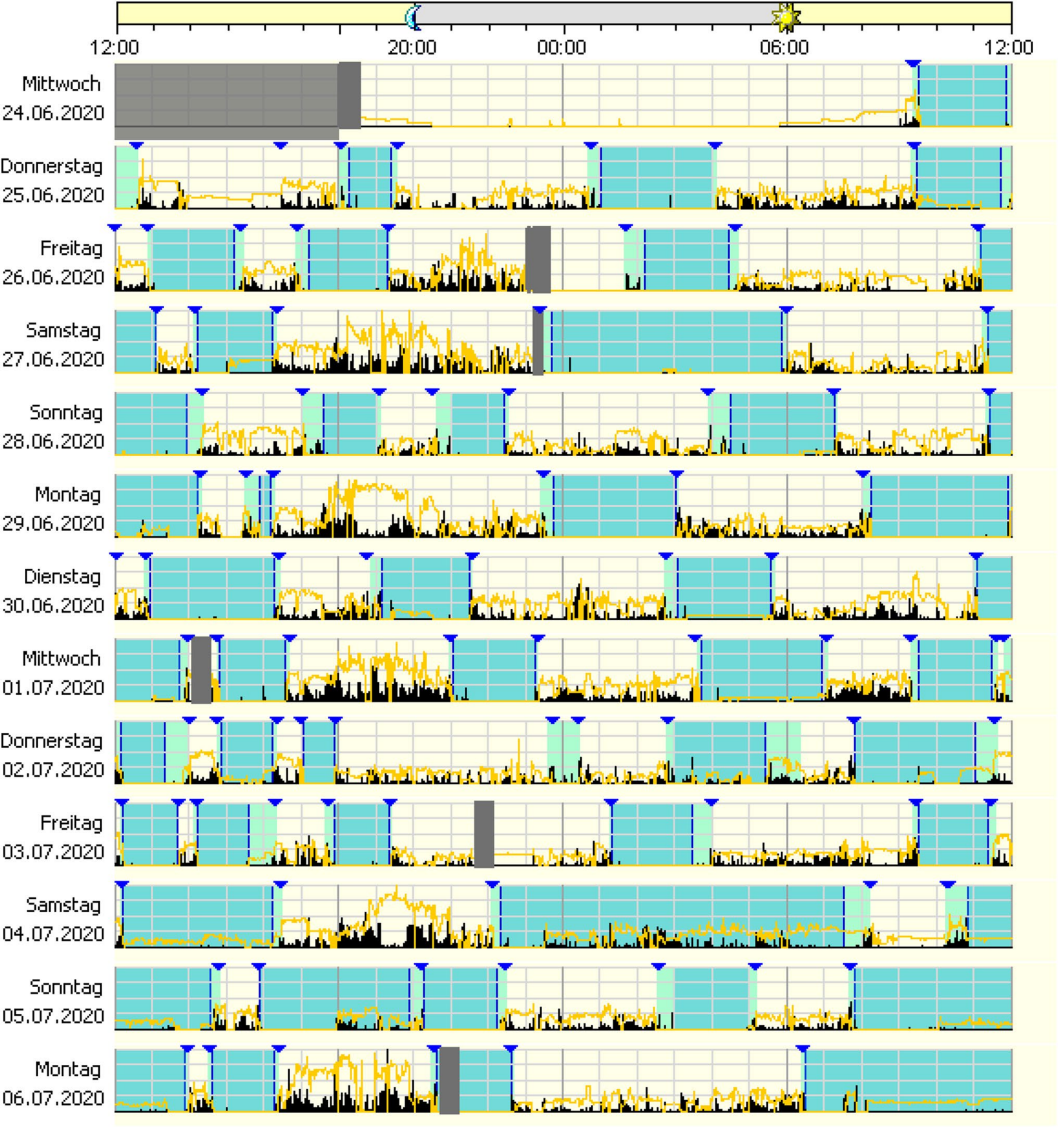
Irregular sleep–wake rhythm due to psychiatric disorder: an example of a wrist actigraphy of a psychiatric out-patient. Treated in a general practitioner setting due to subjective sleep disturbance against a background of depression, addiction with a history of increased alcohol consumption. Hypnotics and drive-enhancing antidepressants were used, and the patient was alcohol-abstinent during the recording period. The actigraphy shows a completely abolished structure of the day-night rhythm, blue triangles corresponding to subjective patient markers corresponding to the limits of bedtime. The continuously recorded activity in the 24-h period appears in black, the light irradiation in yellow. Sleep expert diagnosis: circadian sleep–wake rhythm disorder, an indication for inpatient rhythmic therapy was given

Figure 5 summarises irregular sleep–wake rhythm due to psychiatric disorder.

## Conclusions, expert recommendations in the framework of 3PM

Wrist actigraphy is an important tool for measuring human activity-rest-cycles and recording movements conform with principles of predictive, preventive and personalised medicine, utilising the international classification of sleep disorders (ICSD-3) and following recommendations of AASM.

The paradigm change from reactive to predictive, preventive and personalised medicine (PPPM) is in the focus of the actigraphy applicable to:

- establishing new strategies for increased predictive, preventive and personalised capacity as well as enhanced cost-efficiency of healthcare systems,
- stratifying patients and
- improving individual outcomes at the level of primary, secondary and tertiary care.

Improving physical activity and behaviour patterns have a potential to increase both—the life quality and expectance of healthy as well as physically and/or mentally ill subgroups in the population [111].

Further, in context of participatory medicine, both healthy individuals and patients express their interest in monitoring and improving their behavioural patterns. To satisfy these needs, much equipment is available today, which, however, is not valid for this purpose and can even produce harm to their users. In contrast, wrist actigraphy is a cost-effective validated tool highly recommended and implementable for an individualised diagnostic approach followed by the targeted prevention of a broad spectrum of sleep-related, physical and mental disorders. The approach is also complementary to the personalised treatment of clinically manifested diseases to avoid downstream cascading pathologies and, therefore, improving individual outcomes.

## Data Availability

Not applicable.

## Abbreviations

AASM: American Academy of Sleep Medicine
ASDA: American Sleep Disorders Association
CBT-I: Cognitive behavioural therapy for insomnia
GP: General practitioner
ICU: Intensive care unit
ICSD-3: International classification of sleep disorders 3rd revision
ISWRD: Irregular sleep–wake rhythm disorder
MCI: Mild cognitive impairment
OSA: Obstructive sleep apnea
WHO: World Health Organisation
3PM/PPPM: Predictive preventive personalised medicine
